# Effects of dopexamine on the intestinal microvascular blood flow and leucocyte activation in a sepsis model in rats

**DOI:** 10.1186/cc5011

**Published:** 2006-08-07

**Authors:** Jürgen Birnbaum, Edda Klotz, Claudia D Spies, Björn Lorenz, Patrick Stuebs, Ortrud Vargas Hein, Matthias Gründling, Dragan Pavlovic, Taras Usichenko, Michael Wendt, Wolfgang J Kox, Christian Lehmann

**Affiliations:** 1 Department of Anesthesiology and Intensive Care Medicine, Campus Charité Mitte and Campus Virchow Klinikum, Charité-University Medicine Berlin, Charitéplatz 1, 10117 Berlin, Germany; 2 Department of Anesthesiology and Intensive Care Medicine, Ernst-Moritz-Arndt-University Greifswald, Friedrich Löffler-Str. 23 B, 17475 Greifswald, Germany

## Abstract

**Introduction:**

Dopexamine may be a therapeutic option to improve hepatosplanchnic perfusion in sepsis. To investigate this possibility, we administered dopexamine in an experimental sepsis model in rats.

**Methods:**

This prospective, randomized, controlled laboratory study was conducted in 42 Wistar rats. The animals were divided into three groups. Group 1 served as the control group (CON group). The animals in both groups 2 (LPS group) and 3 (DPX group) received an endotoxin (lipopolysaccharide from *Escherichia coli *– *LPS*) infusion (20 mg/kg for 15 minutes). DPX group additionally received dopexamine (0.5 μg/kg per minute over four hours). One half of the animals in each group underwent studies of intestinal microvascular blood flow (IMBF) using laser Doppler fluxmetry. In the other half an intravital microscopic evaluation of leucocyte-endothelial cell interaction in intestinal microcirculation was conducted. Functional capillary density (FCD) in the intestinal mucosa and in the circular as well as longitudinal muscle layer was estimated.

**Results:**

One hour after endotoxin challenge, IMBF decreased significantly in LPS group to 51% compared with baseline (*P *< 0.05). In DPX group (endotoxin plus dopexamine) we found IMBF values significantly higher than those in LPS group (approximately at the level of controls). The impaired FCD following endotoxin challenge was improved by dopexamine in the longitudinal muscle layer (+33% in DPX group versus LPS group; *P *< 0.05) and in the circular muscle layer (+48% in DPX group versus LPS group; *P *< 0.05). In DPX group, dopexamine administration reduced the number of firmly adherent leucocytes (-31% versus LPS group; *P *< 0.05). Plasma levels of tumour necrosis factor-α were reduced by dopexamine infusion (LPS group: 3637 ± 553 pg/ml; DPX group: 1933 ± 201 pg/ml) one hour after endotoxin challenge.

**Conclusion:**

Dopexamine administration improved IMBF and FCD (markers of intestinal microcirculation) and reduced leucocyte activation (a marker of inflammation) in experimental sepsis.

## Introduction

Sepsis and septic shock represent the most frequent causes of death in surgical intensive care units. Despite an abundance of experimental and clinical studies of sepsis, the mortality rate (40–70%) has remained unchanged over recent years.

Deterioration in hepatosplanchnic perfusion plays a pivotal role in the pathogenesis of sepsis and multisystem organ failure [1,2]. Intestinal hypoperfusion results in a disturbance in mucosal microcirculation, gut barrier dysfunction with increased intestinal permeability, and resulting invasion of bacteria and their toxins into the systemic circulation. Leucocyte-endothelium interactions and cytokine release are signs of the inflammatory reaction [3]. Because of the involvement of impaired hepatosplanchnic perfusion in the pathogenesis of sepsis, maintenance of hepatosplanchnic perfusion is a focus of experimental and clinical sepsis research.

The standard supportive treatment for sepsis consists of ventilatory support, adequate volume resuscitation and application of vasoactive drugs, with the aim being to maintain adequate oxygen delivery to all organs and to the gut in particular. In addition to noradrenaline (norepinephrine), adrenaline (epinephrine), dopamine and dobutamine, dopexamine has been the subject to various investigations [4-6]. Over recent years the influence of synthetic catecholamines – primarily dopexamine – on gastrointestinal microcirculation has come to the fore [7-10]; and what is more, dopexamine also appars to have anti-inflammatory effects [11].

To test the hypothesis that administration of dopexamine can improve parameters of hepatosplanchnic perfusion in experimental endotoxaemia, we used intestinal laser Doppler fluxmetry (LDF) and intravital fluorescence microscopy (IVM). We evaluated the effects of dopexamine on intestinal microvascular blood flow (IMBF; estimated using LDF), on intestinal functional capillary density (FCD), and on leukocyte-venular endothelium interactions (estimated using IVM) in endotoxaemic animals.

## Materials and methods

### Animals

We obtained 42 male Wistar rats (weight 200–250 g, age 6–8 weeks) from Tierzucht Schönwalde GmbH (Schönwalde, Germany). They were housed in chip-bedded cages in air-conditioned animal quarters, and were acclimatized to the institutional animal care unit for one week before the experiments were conducted. The animals were maintained on a 12-hour light/dark cycle and were given free access to water (drinking bottle) and standard rat chow (Altromin^®^; Altromin, Lage, Germany). Food was withdrawn 18 hours before each experiment, whereas water remained freely accessible. Animal experiments were approved by our institutional review board for the care of animals and were performed in accordance with German legislation on protection of animals.

### Anaesthesia and monitoring

The animals were initially anaesthetized with 60 mg/kg pentobarbital (Sigma, Deisenhofen, Germany) intraperitoneally and were supplemented with 20 mg/kg per hour pentobarbital intravenously during the experiment. The animals were fixed in supine position on a heating pad, maintaining a rectal temperature between 36.5°C (97.7°F) and 37°C (98.6°F). Tracheostomy was performed to maintain airway patency, and the animals breathed room air spontaneously. The left jugular vein and carotid artery were cannulated with polyethylene catheters (PE50; inner diameter 0.58 mm; outer diameter 0.96 mm; Portex, Hythe, Kent, UK). The arterial pressure and heart rate were recorded continuously (Biomonitor BMT 5231; RFT, Staßfurt, Germany). The animals received 7.5 ml/kg per hour crystalloid solution (Thomaejonin^®^; Thomae, Biberach, Germany).

### General protocol

The experiments started 30 minutes after cannulation (baseline; time point 0 h). The rats were divided into three groups of 14 animals each. Animals in group 1 did not receive endotoxin and served as controls (CON group). In groups 2 (LPS group) and 3 (DPX group) endotoxaemia was induced by continuous infusion of 20 mg/kg lipopolysaccharide (LPS) from *Escherichia coli*, serotype O55:B5 (Sigma) over 15 minutes. The animals in CON group were administered an equivalent amount of normal saline. Then, animals in DPX group were also administered 0.5 μg/kg per minute dopexamine (Dopacard^®^; Elan Pharma, Munich, Germany) over the four-hour period of observation, which began after completion of the endotoxin infusion. Animals in CON group and in LPS group were given an equivalent amount of normal saline.

In one half of the animals of each group, LDF was performed. The other half of the animals underwent examination of leucocyte adherence on submucosal venular endothelium by IVM of the small bowel wall; they also underwent evaluation of FCD in the intestinal mucosa and the circular as well as longitudinal muscle layers. Measurements of IMBF by LDF were performed at 0, 1, 2 and 4 hours after the start of the experiment. IVM was performed after two hours. Laparotomy for IVM was performed before the start of the endotoxin or placebo infusion. The abdomen was opened by a midline incision. A section of the distal small intestine (10 mm orally from the ileocaecal valve) was placed carefully on a specially designed stage attached to the microscope. During the entire *in vivo *microscopic procedure, intestine was superfused with thermostatically controlled (37°C [98.6°F]) crystalloid solution (Thomaejonin^®^) in order to avoid drying and exposure to ambient air [12]. At the end of the experiments, the animals were euthanized by pentobarbital overdose.

### Laser Doppler fluxmetry

The glass fibre laser Doppler probe (diameter 120 μm, wave length 810 nm, resulting penetration depth about 1–2 mm [13]) was calibrated using a calibration solution (Lawrenz GmbH, Sulzbach, Germany) and attached to a distal ileal segment with enbucrilate (Histoacryl^®^; Braun, Melsungen, Germany) without any compression or traction of the gut. Pilot experiments have demonstrated that low dosages of enbucrilate do not influence intestinal blood flow or intestinal function. The position of the probe was not altered during the course of the experiment. The intestine was neither touched nor moved. A transparent plastic cover was placed over the preparation, which was kept moist throughout the experiment with temperature controlled Ringer's solution (37°C). The probe was connected to a laser blood flow monitor (MBF3D; Moor Instruments, Axminster, UK).

The flux values were calculated after measuring the speed and concentration of the moving red blood cells. The speed was estimated according to the magnitude of the laser Doppler frequency shift, whereas concentration was taken from the total power of the photodetector current. Laser Doppler flux signals were analogue-to-digital (AD) converted and recorded using a personal computer-based system for four minutes, with a sampling rate of 40 Hz. The laser Doppler flux signal was low pass filtered by the MBF3D with a corner frequency of 0.1 Hz before AD conversion in order to avoid aliasing effects at a sampling rate of 40 Hz. The region for measurements was selected by visual control. Regions with larger vessels or peristalsis were avoided. At chosen regions, 100 perfusion units were aspired. Offline, stationarity of the signal was verified by visual inspection of the time series (minimal period 3.5 minutes).

### Intravital microscopy

The intravital fluorescence videomicroscopy was performed by using an epifluorescent microscope (Axiotech Vario, filter block No. 20; Zeiss, Germany) with a 50-W HBO (Osram, Munich, Germany) short arc mercury lamp and equipped with a 10× long distance (10/0.5; Fluar, Zeiss, Oberkochen, Germany) and a 20× water immersion (20/0.5; Achroplan, Zeiss) objective (mesentery: 40× water immersion, 40/0.8; Achroplan, Zeiss) and a 10× eyepiece. The images were transferred to a monitor (LDH 2106/00; Philips Electronics, Eindhoven, The Netherlands) with the help of a video camera (FK 6990-IQ; Pieper, Schwerte, Germany) and were recorded at the same time on a videotape using a video casette recorder (Panasonic AG 6200; Matsushita, Osaka, Japan) for offline evaluation.

The leucocytes were stained *in vivo *by intravenous injection of 0.2 ml of 0.017 g % rhodamine 6G (MW 479; Sigma, Deisenhofen, Germany) for contrast enhancement, enabling visualization in the microvasculature. The microvessels in the intestinal submucosal layer were classified by their order of branching, in accordance with the classification proposed by Gore and Bohlen [14]. Submucosal collecting venules (V1) as well as postcapillary venules (V3) were analyzed. Activated leucocytes, adhering firmly to the venular endothelium, were defined in each vessel segment as cells that did not move or detach from the endothelial lining within an observation period of 30 s. They are indicated as number of cells per square millimetre of endothelial surface, calculated from diameter and length of the vessel segment studied, assuming cylindrical geometry. Seven vessels of each population were evaluated in every animal. The evaluation of leucocyte adherence was performed in a blinded manner.

After two hours of endotoxaemia, 50 mg/kg body weight FITC-labeled bovine serum albumen (Sigma) was administered intravenously to distinguish plasma from red blood cells (negative contrast). The assessment of FCD in the intestinal mucosa and the circular as well as longitudinal muscle layers was performed by morphometric determination of the length of red blood cell perfused capillaries per area, in accordance with the method proposed by Schmid-Schönbein and colleagues [15]. Five separate fields were examined in each layer.

### Tumour necrosis factor-α

At baseline (0 hours) and after 1, 2 and 4 hours, 200 μl heparinized arterial blood samples were drawn for estimation of plsma levels of tumour necrosis factor (TNF)-α. For analysis we used a rat-specific solid-phase enzyme-linked immunosorbent assay kit (Genzyme Corp., Cambridge, MA, USA) employing the multiple antibody sandwich principle in accordance with the manufactorer's instructions. A microtitre plate, pre-coated with monoclonal anti-TNF-α, was used to capture rat TNF-α from test samples. Unbound material was removed by washing with buffer solution. A peroxidase-conjugated polyclonal anti-TNF-α antibody, which binds to captured rat TNF-α, was added. By addition of substrate solution, a peroxidase catalyzed colour change proceeds and the absorbence measured at 450 nm is proportional to the concentration of rat TNF-α in the sample. A standard curve was obtained by plotting the concentrations of rat TNF-α standards versus their absorbences. The TNF-α concentration of the samples was determined using this standard curve. Intra-assay reproducibility is indicated by the following coefficients of variation: at rat TNF-α mass 1,024.4 pg/ml the coefficient was 6.6, and at rat TNF-α mass of 376.5 pg/ml it was 3.7. The inter-assay reproducibility is indicated by the following coefficients of variation: at rat TNF-α mass 766.4 pg/ml the coefficient was 3.8, and at a rat TNF-α mass of 168.3 pg/ml it was 6.5.

### Statistical analysis

The data analysis was performed by means of a statistical software package (SigmaStat; Jandel Scientific, Erkrath, Germany). All data were expressed as group mean ± standard error of the mean. After establishing that the data conformed with tests of normality of distribution and equality of variance, they were analyzed using one-way analysis of variance followed by Scheffé's test. *P *< 0.05 value was considered statistically significant.

## Results

None of the animals died during the period of observation.

### Heart rate and mean arterial pressure

In both endotoxaemic groups (LPS and DPX group), endotoxin challenge resulted in increased heart rate and decreased mean arterial pressure (MAP) compared with baseline and compared with controls (Table 1). One hour after endotoxin challenge, MAP levels in DPX group were restored to control levels and remained at this level.

### Laser Doppler fluxmetry

**Figure 1:**
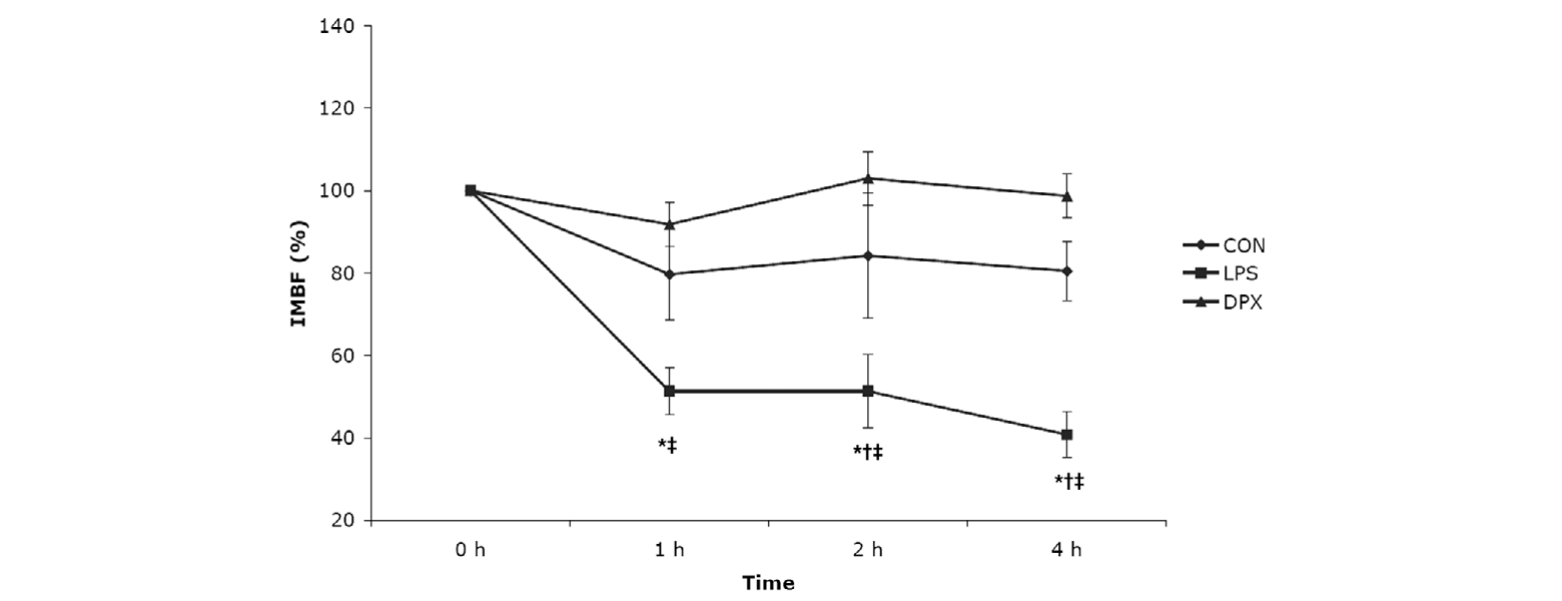
Intestinal microvascular blood flow. Shown is intestinal microvascular blood flow (IMBF) as a percentage of baseline; measurements taken at baseline (time point 0 hours) and at 1, 2 and 4 hours after the start of the experiment. CON, control group; DPX, DPX group (endotoxin plus dopexamine); LPS, LPS group (endotoxin infusion only). *P < 0.05 versus baseline; ^†^*P *< 0.05 versus CON; ^‡^*P *< 0.05 versus DPX.

One hour after endotoxin challenge IMBF decreased significantly in LPS group to 51% compared with baseline (*P *< 0.05; Figure 1). At two and four hours after the start of the experiment, we also observed decreased laser Doppler flow in LPS group compared with that in CON group. Animals in DPX group exhibited significantly higher values compared with those in LPS group.

### Functional capillary density

**Figure 2:**
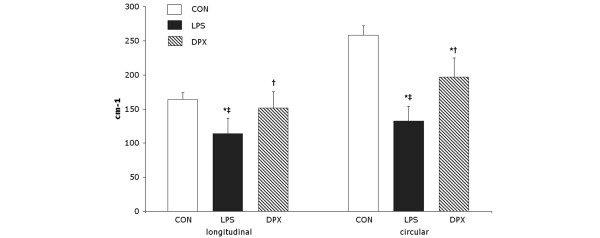
Functional capillary density. Shown is functional capillary density (FCD) in the longitudinal and circular muscularis layers; measurements were taken at two hours after the start of endotoxaemia. CON, control group; DPX, DPX group (endotoxin plus dopexamine); LPS, LPS group (endotoxin infusion only). *P < 0.05 versus CON; ^†^*P *< 0.05 versus LPS; ^‡^*P *< 0.05 versus DPX.

The impairment in FCD due to endotoxin challenge was prevented by dopexamine in the longitudinal muscle layer (+33% in DPX group versus LPS group; *P *< 0.05) and was also attenuated in the circular muscle layer (+48% in DPX group versus LPS group; *P *< 0.05; Figure 2). FCD in the intestinal mucosa was not influenced either by endotoxin challenge or by dopexamine (data not shown).

### Leucocyte-endothelium interaction

**Figure 3:**
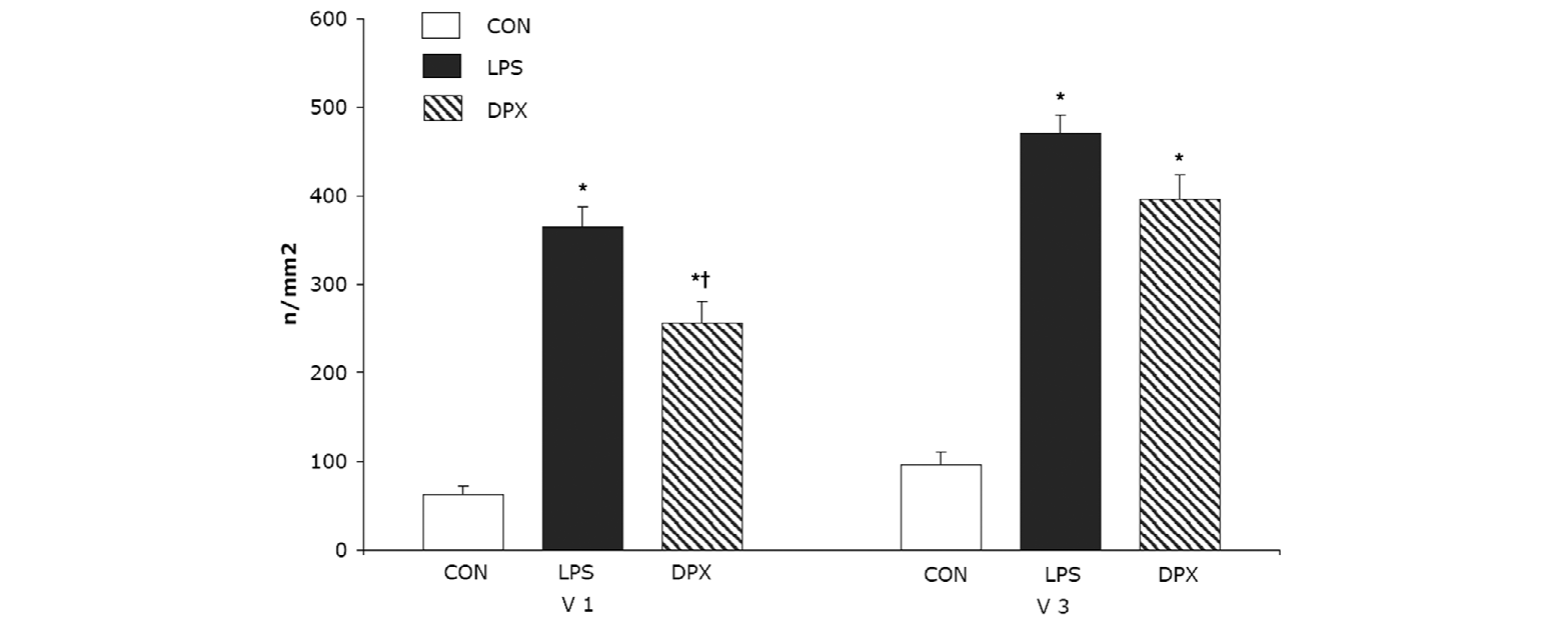
Firmly adherent leucocyte count. Shown are the counts of firmly adherent leucocytes (sticker) in V1 and V3 venules; measurements were taken two hours after endotoxin challenge. CON, control group; DPX, DPX group, (endotoxin plus dopexamine); LPS, LPS group (endotoxin infusion only). *P < 0.05 versus CON; ^†^*P *< 0.05 versus LPS.

Figure 3 summarizes counts of firmly adherent leucocytes in V1 and V3 venules of intestinal submucosa two hours after the start of the endotoxin challenge. In V1 venules the count was sixfold higher after endotoxin challenge in LPS group compared with CON group (LPS group 364 ± 23 per mm^2 ^versus CON group 62 ± 10 per mm^2^; *P *< 0.05). In V3 venules endotoxin administration in LPS group resulted in a fivefold increase in adherent leucocytes compared with controls (470 ± 21 per mm^2 ^versus 96 ± 14 per mm^2^; *P *< 0.05).

In DPX group, we found a significant reduction in endotoxin-induced leucocyte adherence (-31%) in the V1 subpopulation of venules relative to that in LPS group (*P *< 0.05). In V3 venules the reduction in leucocyte adherence (-16%) did not achieve statistical significance. Endotoxin challenge resulted in a decrease in leucocyte rolling to 23% compared with CON group in V1 venules and to 12% in V3 venules (*P *< 0.05). This decrease was not influenced by dopexamine administration.

### Tumour necrosis factor-α

**Figure 4:**
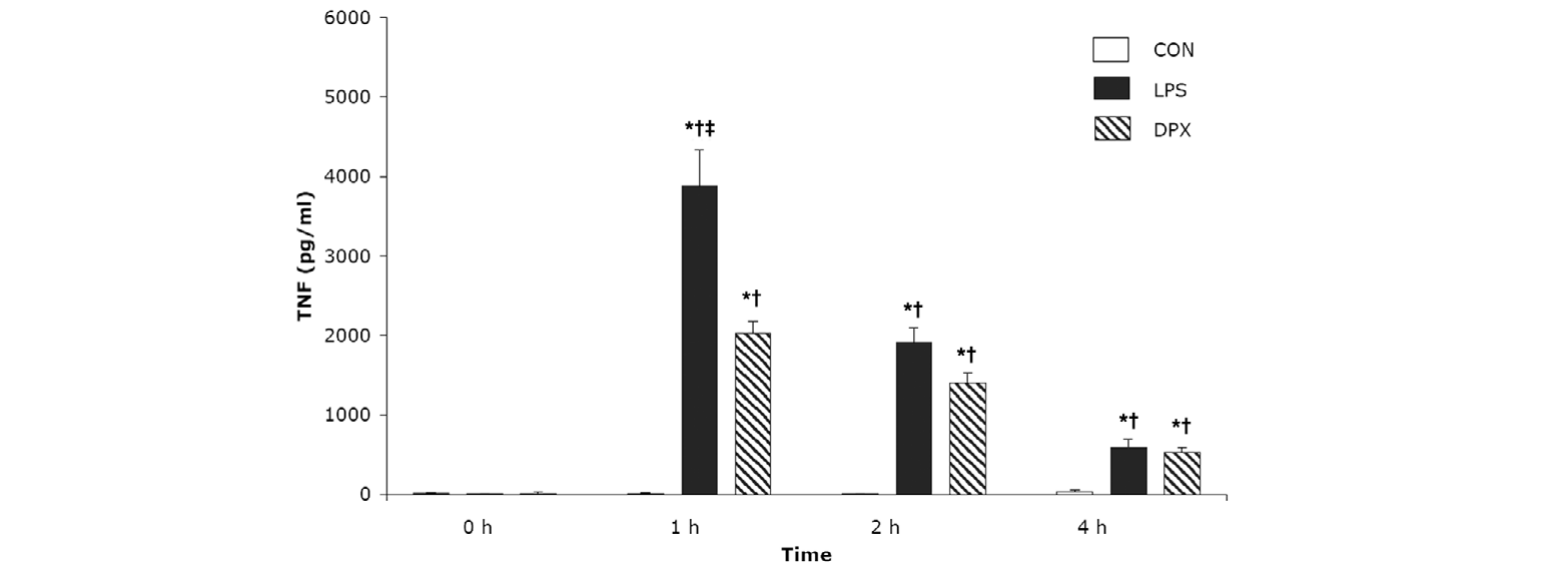
Tumour necrosis factor-α levels. Shown are tumour necrosis factor (TNF)-α levels; measurements were taken one, two and four hours after induction of endotoxaemia. CON, control group; DPX, DPX group (endotoxin plus dopexamine); LPS, LPS group (endotoxin infusion only). *P < 0.05 versus baseline; ^†^*P *< 0.05 versus CON; ^‡^*P *< 0.05 versus DPX.

One hour after the start of endotoxin challenge, we identified the highest TNF-α levels in LPS group (Figure 4). Dopexamine administration significantly reduced TNF-α levels at this time point (3,637 ± 553 pg/ml in LPS group; 1,933 ± 201 pg/ml in DPX group).

## Discussion

In the present study, the endotoxin challenge induced a dramatic decrease in IMBF. This is in accordance with the results of our previous investigations in rats [16]. The administration of dopexamine significantly increased IMBF at all measurement times. Similar increases in IMBF, measured using LDF, during dopexamine administration have been demonstrated in other experimental and clinical settings [17-19]. In a mild hypothermic cardiopulmonary bypass model in rabbits, dopexamine significantly increased jejunum and ileum blood flow, estimated using LDF [17]. In an experimental setting in pigs, jejunal mucosal blood flow was not influenced by dopexamine infusion during intestinal hypotension, but dopexamine brought about intestinal vasodilatation [10]. In postoperative cardiosurgical patients dopexamine increased jejunal mucosal perfusion by 20%, as measured using endoluminal LDF [7]. In patients in septic shock, a combination of dopexamine and noradrenaline enhanced gastric mucosal blood flow (estimated using LDF) to an extent greater than that with adrenaline alone, and the authors concluded that this combination could be an interesting option in the treatment of septic shock [20]. On the other hand, dopexamine was unable to improve gastric intramucosal partial carbon dioxide tension [21] and could not enhance haemodynamic function and tissue oxygenation [22] during major abdominal surgery.

In addition, the FCD in the longitudinal and circular muscle layers – a marker of microcirculation – was impaired in endotoxaemic animals, as expected. Dopexamine administration led to attenuation of this microcirculatory disturbance. However, neither endotoxin nor dopexamine had any influence on FCD in intestinal mucosa. At first glance, the unchanged FCD in the intestinal mucosa appears to be contradictory to the changes in IMBF in the intestinal wall. To understand this phenomenon, it is important to take into consideration the fact that, because of the laser penetration depth of about 1–2 mm, IMBF reflects the blood flow in the whole gut wall. In contrast, FCD reflects only the perfusion of the capillaries of the focused layer. Moreover, FCD is not diminished when blood flow in capillaries is lower, but only when capillaries are occluded completely. Another explanation could be a redistribution of blood flow within the intestinal wall.

Animals of all groups received fluid resuscitation of 7.5 ml/kg per hour crystalloid solution. After endotoxin challenge, heart rate was increased and MAP was decreased in the endotoxaemic groups (LPS group and DPX group; Table 1). In CON group in particular, MAP was stable during the trial. Nevertheless, intravascular hypovolaemia can not be excluded and is a typical occurrence in sepsis. The aim of this model is to induce manifestations that are characteristic of sepsis. Because the animals in the two endotoxaemic groups were treated in an identical manner (with the exception of dopexamine treatment in DPX group), the differences between the two groups of septic animals must result from dopexamine administration.

To interpret the results of the study it is important to be aware of some limitations of the setting. We cannot exclude an influence of hypovolaemia on the results, although this is a typical phenomenon in sepsis. Cardiac output and global splanchnic blood flow were not measured in the model but they could provide more data that may help in interpreting the results and appreciating the effect of dopexamine.

In the study we found a significant reduction in activated leucocytes adhering firmly to the endothelium in dopexamine-treated endotoxaemic animals. IVM is a standard method used in *in vivo *studies of microcirculation [23]. Dynamic processes such as interactions between leucocytes and endothelium, as well as perfusion of capillaries, are visible [24]. The adherence of leucocytes in endotoxaemia is a multistep process. After the increase in margination of leucocytes from the centre of the bloodstream, cells are temporarily adherent (rolling) to the endothelium of the vessel wall [25,26]. The next step is firm adherence of leucocytes to endothelium [27]. Activated leucocytes release various mediators, including oxygen free radicals, elastase, collagenase and myeloperoxidase. This leads to an increase in endothelium permeability and to activation of other cascade systems [28-30]. The emigration of leucocytes represents the last step in leucocyte activation. Tissue damage to vascular endothelium is one of the consequences of leucocyte activation [31]. Decreasing or inhibiting leucocyte adherence may be a beneficial therapeutic approach in endotoxaemia and sepsis.

In the present study we found a fivefold to sixfold higher count of adherent leucocytes, depending on the size of venules, following endotoxin challenge compared with control animals. The improvement in microcirculation attributable to dopexamine, as indicated by the increase in LDF, appears to have an important influence on leucocyte adherence. The adherence also depends on shear stress in the blood flow. The restoration of normal blood flow diminishes the interaction between leucocyte and endothelium [27]. In a similar experimental setting, dopexamine reduced leucocyte adherence in mesenteric vessels in endotoxaemia [32]. The antioxidative effects of dopexamine may also be responsible for the reduction in leucocyte adherence. After experimental endotoxin challenge, production of uric acid was reduced by dopexamine infusion [33]. As a result of decreased radical formation in the xanthine oxidase pathway, this may lead to reduced leucocyte-endothelium interaction.

TNF-α is an initial marker of sepsis. In experimental endotoxaemia it is detectable within a few minutes. Depending on the dose of endotoxin administered, TNF-α levels increase as soon as after 1–2 hours after endotoxin challenge [34-36]. Hence, TNF-α is a valuable indicator of sepsis induction in experimental settings. After one hour we found peak levels of TNF-α indicating effective induction of endotoxaemia. After two hours TNF-α decreased to 50% of the level at one hour. At four hours TNF-α levels were also increased compared with baseline. The degree of TNF-α release in the present study is comparable to the findings of others [37-39]. In the animals of the control group we found TNF-α levels to be exclusively in the lower range of 50 pg/ml. Thus, the inflammatory response is not a result of preparations before the start of the experiment (insertion of catheters, laparotomy, among other factors). The dopexamine infusion significantly reduced the release of TNF-α. In endotoxaemic animals treated with dopexamine we found reductions in TNF-α release of 47% at one hour and 30% at two hours after endotoxin challenge compared with untreated endotoxaemic animals. In patients the increase in TNF-α levels after cardiopulmonary bypass was attenuated by dopexamine application [11]. Dopexamine had no effect on splanchnic blood flow.

In our model, we performed no dose-response studies to find out whether other doses of dopexamine were more effective. In order to elucidate the effects of dopexamine in clinical sepsis, additional studies in patients are required.

## Conclusion

The administration of dopexamine improved IMBF and FCD (markers of intestinal microcirculation) and reduced leucocyte activation (a marker of inflammation) in experimental sepsis.

## Key messages

• Dopexamine improved intestinal microvascular blood flow in a sepsis model in rats.

• Dopexamine reduced endotoxin-induced leucocyte adherence in venules of the intestinal submucosa.

• Dopexamine infusion significantly reduced release of TNF-α, which is an early marker of sepsis.

## Abbreviations

AD = analogue-to-digital; CON = control group; DPX = DPX group (endotoxin plus dopexamine); FCD = functional capillary density; IMBF = intestinal microvascular blood flow; IVM = intravital microscopy; LDF = laser Doppler fluxmetry; LPS = LPS group (endotoxin infusion only); MAP = mean arterial pressure; TNF = tumour necrosis factor.

## Competing interests

The authors declare that they have no competing interests.

## Authors' contributions

JB, EK, CS and ChL coordinated the study and drafted the manuscript. ChL, BL and PS performed the IVM and collected the data. OVH, MG, DP, TU and MW helped to draft the manuscript. CS, WJK and ChL conceived and designed the study, and performed the statistical analysis.

## Figures and Tables

**Table 1:** Heart rate and mean arterial pressure findings

	Group	Time (hours)					
		
		0	0.5	1	2	3	4
Heart rate (beats/minute)	CON	335 ± 18	347 ± 20	351 ± 20	349 ± 21	343 ± 19	339 ± 9*^†^
	LPS	342 ± 11	347 ± 12	365 ± 17	387 ± 11^‡^	378 ± 11^‡^	390 ± 9^‡^
	DPX	321 ± 9	366 ± 9	399 ± 7^§^	394 ± 8^§^	384 ± 15^§^	394 ± 12^§^
Mean arterial pressure (mmHg)	CON	120 ± 5	119 ± 8*†	109 ± 7*	107 ± 8	116 ± 3	118 ± 5*
	LPS	116 ± 3	74 ± 2^‡^	87 ± 3^‡^	94 ± 4^‡^	88 ± 3^‡^	89 ± 5^‡^
	DPX	120 ± 6	70 ± 2^§^	107 ± 7^§^	102 ± 7^§^	104 ± 8^§^	99 ± 9^§^

CON, control group; DPX, DPX group (endotoxin plus dopexamine); LPS, LPS group (endotoxin infusion only). *P < 0.05 CON versus LPS; ^†^*P *< 0.05 CON versus DPX; ^‡^*P *< 0.05 LPS versus LPS at baseline; ^§^*P *< 0.05 DPX versus DPX at baseline.
